# P72 Optimizing empirical antibiotic prescribing in neonatal sepsis: global review of clinical factors and stewardship implementation

**DOI:** 10.1093/jacamr/dlag102.078

**Published:** 2026-06-26

**Authors:** Hafsah Shabir, Rasha Abdelsalam Elshenawy

**Affiliations:** School of Health, Medicine and Life Sciences, University of Hertfordshire, College Lane, Hatfield AL10 9AB, UK; School of Health, Medicine and Life Sciences, University of Hertfordshire, College Lane, Hatfield AL10 9AB, UK

## Abstract

**Background:**

Neonatal sepsis is a leading cause of neonatal mortality worldwide, affecting approximately three million neonates annually and accounting for an estimated 15% of all neonatal deaths.^1^ Sepsis is classified by onset: early-onset sepsis (EOS) occurs within the first 72 h of life, while late-onset sepsis (LOS) presents after 72 h. Clinical diagnosis is challenging, as presenting features are frequently non-specific. Diagnostic capacity varies considerably between high-income countries (HICs) and low- and middle-income countries (LMICs), and empirical antibiotic prescribing practice differs accordingly.^2^ Escalating antimicrobial resistance (AMR) further complicates treatment decisions.

**Objectives:**

To identify clinical, laboratory, and microbiological variables influencing empirical antibiotic selection in neonatal sepsis; assess their association with treatment outcomes; and evaluate antimicrobial stewardship interventions across HIC and LMIC settings.

**Methods:**

A systematic search was conducted across OVID MEDLINE, Cochrane Library, and Scopus to identify studies published between 2016 and 2026. Studies reporting clinical, laboratory, or microbiological variables used in the diagnosis and treatment of confirmed or suspected neonatal sepsis (early-onset or late-onset) were included using a PICO framework. A two-stage screening process was applied, comprising title and abstract review followed by full-text assessment. Data were extracted across seven domains: study characteristics, neonatal demographics, clinical presentation, laboratory parameters, microbiology, antibiotic prescribing, and outcomes. The WHO AWaRe 2022 classification was applied to all reported antibiotic agents. Quantitative data analysis was performed descriptively. Ethical approval was not required, as no primary data were collected.

**Results:**

Database searching yielded 972 records, of which 12 studies met the inclusion criteria, comprising 22 709 participants across 17 countries in Asia, Africa, Europe, and South America. Late-onset sepsis predominated in LMIC settings (77.4%), while early-onset sepsis predominated in HIC settings (74.9%), with direct implications for empirical regimen selection. Ampicillin and gentamicin, both Access class agents, were the most frequently prescribed empirical antibiotics, each reported in 50% of included studies. Watch class antibiotics featured in 47% of study arms, most commonly piperacillin/tazobactam (25%), reflecting the MDR Gram-negative burden in LMIC neonatal intensive care units. Antibiotic escalation occurred in 25% of neonates, while de-escalation was recorded in only 5.9%, representing a significant stewardship opportunity. *Klebsiella* spp. was the dominant pathogen (29.2%), followed by coagulase-negative staphylococci (11.2%) and *Escherichia coli* (9.8%). Antimicrobial resistance rates were highest for ampicillin (100%), gentamicin and third-generation cephalosporins (77% each), with meropenem resistance at 35%, rendering the WHO first-line regimen largely ineffective across LMIC settings.

**Conclusions:**

This study establishes a robust evidence base for improving empirical antibiotic prescribing in neonatal sepsis across diverse global settings. Findings highlight the urgent opportunity to develop setting-specific prescribing guidelines that reflect local pathogen distribution and resistance profiles. Shorter antibiotic courses demonstrated non-inferior outcomes, supporting their adoption as a practical stewardship strategy. Investment in LMIC diagnostic capacity and structured antimicrobial stewardship programmes presents a significant opportunity to optimize neonatal outcomes, improve antibiotic prescribing, preserve antibiotic efficacy, and meaningfully reduce the global burden of AMR.
